# Hypoxia increases expression of selected blood–brain barrier transporters GLUT-1, P-gp, SLC7A5 and TFRC, while maintaining barrier integrity, in brain capillary endothelial monolayers

**DOI:** 10.1186/s12987-021-00297-6

**Published:** 2022-01-04

**Authors:** Burak Ozgür, Hans Christian Cederberg Helms, Erica Tornabene, Birger Brodin

**Affiliations:** Department of Pharmacy, University of Copenhagen, Universitetsparken 2, 2100 Copenhagen, Denmark

**Keywords:** Angiogenesis, Hypoxia, Low oxygen tension, Endothelial cells, Blood–brain barrier, Tight junctions, Brain

## Abstract

**Background:**

Brain capillary endothelial cells (BCECs) experience hypoxic conditions during early brain development. The newly formed capillaries are tight and functional before astrocytes and pericytes join the capillaries and establish the neurovascular unit. Brain endothelial cell phenotype markers P-gp (ABCB1), LAT-1(SLC7A5), GLUT-1(SLC2A1), and TFR(TFRC) have all been described to be hypoxia sensitive. Therefore, we hypothesized that monolayers of BCECs, cultured under hypoxic conditions, would show an increase in LAT-1, GLUT-1 and TFR expression and display tight endothelial barriers.

**Methods and results:**

Primary bovine BCECs were cultured under normoxic and hypoxic conditions. Chronic hypoxia induced HIF-1α stabilization and translocation to the nucleus, as judged by immunocytochemistry and confocal laser scanning imaging. Endothelial cell morphology, claudin-5 and ZO-1 localization and barrier integrity were unaffected by hypoxia, indicating that the tight junctions in the BBB model were not compromised. SLC7A5, SLC2A1, and TFRC-mRNA levels were increased in hypoxic cultures, while ABCB1 remained unchanged as shown by real-time qPCR. P-gp, TfR and GLUT-1 were found to be significantly increased at protein levels. An increase in uptake of [^3^H]-glucose was demonstrated, while a non-significant increase in the efflux ratio of the P-gp substrate [3H]-digoxin was observed in hypoxic cells. No changes were observed in functional LAT-1 as judged by uptake studies of [^3^H]-leucine. Stabilization of HIF-1α under normoxic conditions with desferrioxamine (DFO) mimicked the effects of hypoxia on endothelial cells. Furthermore, low concentrations of DFO caused an increase in transendothelial electrical resistance (TEER), suggesting that a slight activation of the HIF-1α system may actually increase brain endothelial monolayer tightness. Moreover, exposure of confluent monolayers to hypoxia resulted in markedly increase in TEER after 24 and 48 h, which corresponded to a higher transcript level of CLDN5.

**Conclusions:**

Our findings collectively suggest that hypoxic conditions increase some BBB transporters' expression via HIF-1α stabilization, without compromising monolayer integrity. This may in part explain why brain capillaries show early maturation, in terms of barrier tightness and protein expression, during embryogenesis, and provides a novel methodological tool for optimal brain endothelial culture.

**Supplementary Information:**

The online version contains supplementary material available at 10.1186/s12987-021-00297-6.

## Introduction

The brain capillary endothelium is the major interface for exchanging molecules between the systemic circulation and the brain parenchyma. The capillaries also serve to restrict the blood-to-brain passage of a wide range of endogenous compounds and xenobiotics [1], the "blood–brain barrier" (BBB) function. The mechanistic basis for the barrier function is the physical tightness of the brain endothelium ensured by tight junction (TJ) complexes, with a high expression of the low-molecular-weight impermeable claudin-5 (Cl-5, CLDN5) along with the expression of a range of efflux transporters, with P-glycoprotein (P-gp, ABCB1) and breast cancer-related protein (BCRP, ABCG2) as the most prominent [2, 3]. Lack of fenestration, reduced pinocytotic activity, and the presence of metabolizing enzymes also contribute to the low barrier permeability [2, 4]. The brain capillary endothelium is surrounded by pericytes and astrocytic end-feet processes that regulate and maintain the phenotype of the endothelium [5]. The functional complex of capillaries associated with astrocytes (connected to neurons), pericytes and microglia, is termed the neurovascular unit (NVU).

Our knowledge on the effects of hypoxia on brain capillary endothelial cell monolayers is still limited. Hypoxia may regulate the expression levels of TJ proteins, solute carriers, efflux transporters and receptors for nutrients and hormones, thus compromising barrier function. Studies on the effects of hypoxia on brain endothelial cells are sparse, but some in vitro studies on cultured brain endothelial cells indicate that hypoxia causes disruption and opening of TJs, resulting in barrier breakdown [6–8]. However, evidence also suggests that hypoxia is present in the embryonic neuroectoderm undergoing early brain vascularization [9–11]. Early in brain development when capillaries enter the hypoxic brain parenchyma, they grow into tight and functional monolayers with the expression of brain capillary endothelial marker proteins before the astrocytes and pericytes are recruited, and the mature neurovascular unit is formed [2, 12–14].

Cells respond to hypoxia primarily through hypoxia-inducible factor-1 (HIF-1), which is comprised of an oxygen tension-controlled alpha subunit (HIF-1α) and a constitutively expressed HIF-1β subunit [15]. Under low oxygen conditions, HIF-1α is stabilized and translocated to the nucleus, where it regulates several genes, including those involved in angiogenesis, cell proliferation, and apoptosis.

Early vascularization in the central nervous system (CNS) begins with angiogenesis when capillaries sprouting from existing periphery vessels grow into the avascular neuroectoderm and form intraneural vessels [16], presumably guided by the low oxygen tension [9]. This process is followed by the formation of intracerebral branches characterized by high endothelial cell proliferation [16]. TJs are apparently expressed in early brain vascularization, and the capillaries act as a physical barrier against proteins and small molecules [2, 13, 14]. Furthermore, it has been observed that hypoxia facilitates vascular differentiation of pluripotent stem cells [17]. Therefore, we hypothesized that a hypoxic environment could influence transporter expression and barrier integrity in brain capillary endothelial cells.

In brief, we cultured primary bovine brain capillary endothelial cells (BCECs) under hypoxic conditions and investigated the expression of selected transporters reported to be hypoxia sensitive in other model systems, as well as barrier integrity. We observed that the hypoxic conditions (1% O_2_,) stabilized HIF-1α, led to upregulation of specific BBB transporter systems at both the transcriptomic and protein level. The upregulations were found to be HIF-1α-dependent. Furthermore, the hypoxic conditions did not affect the localization of the tight junction proteins Cl-5 and ZO-1 or monolayer integrity as measured by TEER measurements. These results demonstrate that hypoxia may actually participate in generating a mature brain endothelial phenotype, knowledge which may be of use for understanding the role of hypoxia in early brain development and in brain endothelial cell culture studies.

## Materials and methods

All chemicals and reagents were obtained from Sigma-Aldrich (Brondby, Denmark) unless otherwise stated.

### Isolation of bovine brain capillaries

Bovine brains from < 12-month-old calves were obtained from a local abattoir (Mogens Nielsens Kreaturslagteri, Herlufmagle Denmark) and the brain capillaries isolated as previously described by Helms and Brodin [18]. Briefly, the meninges were removed, and the gray matter from the cortices scraped off, collected in Dulbecco's Modified Eagle Medium (DMEM), and homogenized in a Dounce tissue grinder (Wheaton Science Products, Millville, USA). The homogenates were filtered using 160-µm nylon net filters, and the capillary fragments collected by flushing the filters with DMEM supplemented with 10% fetal bovine serum (FBS), 10 mL∙L^−1^ MEM non-essential amino acids (NEAAs), and 100 U∙L^−1^ – 100 µg∙mL^−1^ penicillin–streptomycin (Pen-Strep). The collected capillary fragments were centrifuged for 5 min at 500 g twice. The resulting pellets were resuspended in digestive enzyme mix containing DNase I (170 U∙mL^−1^), collagenase type III (200 U∙mL^−1^), and trypsin TRL (90 U mL^−1^) and incubated for 60–75 min at 37 °C. This was followed by filtration using 200-µm nylon net filters and centrifugation for 5 min at 500 g. The isolated capillary fragments were resuspended in freezing medium containing 90% FBS and 10% dimethyl sulfoxide (DMSO), aliquoted in cryovials, and cryopreserved for later use.

### Bovine endothelial cell culture

Primary brain capillaries were seeded on pre-coated (collagen IV and fibronectin) T75 flasks and cultured in DMEM supplemented with 10% FBS, 10 mL∙L^−1^ NEAAs, 100 U∙L^−1^ – 100 µg∙mL^−1^ Pen-Strep, and 125 µg/mL heparin (DMEM-Comp) for four days at 37 °C in 10% CO_2_. In the first two days, DMEM-Comp was supplemented with 4 μg/mL of puromycin. After four days of culture, the outgrown endothelial cells (approximately 80% confluency) were washed twice in PBS and passaged by trypsinization (~ 2 min) with trypsin/EDTA. The cells were subsequently seeded on collagen IV and fibronectin-coated Transwell inserts (surface area = 1.12 cm^2^, pore size = 0.4 μm, Corning, Schiphol, the Netherlands) at a density of 90,000 cells ∙ cm^−2^. The cells were cultured in DMEM-Comp medium under normoxic (90% room air-10% CO_2_, 37 °C) or hypoxic (1% O_2_, 10% CO_2_ and 89% N_2_, 37 °C) conditions in a hypoxia workbench (X3 Xvivo System, BioSpherix, NY, USA). After three days of culture, DMEM-Comp was replaced with differentiation medium (DMEM-TES) consisting of DMEM supplemented with 8-(4-CPT)-cyclic adenosine monophosphate (312.5 μM), dexamethasone (0.5 μM), RO-20–1724 (17.5 μM), and N-[tris(hydroxymethyl)methyl]-2-aminoethanesulfonic acid (TES; 50 mM), and the cells cultured for an additional three days under normoxic and hypoxic conditions. DMEM-Comp and DMEM-TES were supplemented with desferrioxamine (DFO) at 10, 20, 50, 100, and 150 µM or with 3-(5′-hydroxymethyl-2′-furyl)-1-benzyl indazole at 1, 2, 5, 10, and 25 µM in studies involving the chemical stabilization and destabilization of HIF-1α.

For hypoxia studies on confluent monolayers, cells were first allowed to grow into confluency under normoxic conditions for six days (three days in DMEM-Comp and three days in DMEM-TES), followed by exposure to hypoxia (1% O_2_) or continued culture under normoxic conditions for 48 h.

### Transendothelial electrical resistance measurements

Transendothelial electrical resistance (TEER) was measured using an EndOhm Chamber connected to an EVOM2 Meter (World Precision Instrument, England, UK). All TEER measurements were performed on Day 6.

### Isolation of mRNA and cDNA synthesis

Total RNA was extracted from the cells from day 6. At least three permeable supports were used to generate each RNA sample. The mRNA was extracted from the cell culture and isolated using the GenElute™ Universal Total RNA Purification Kit according to the manufacturer's instructions. The material was treated using the DNAse I Kit prior to cDNA synthesis. The concentration and purity of the RNA were measured using the NanoDrop 2000c spectrophotometer (Thermo Fisher Scientific). Reverse transcription was performed with 0.5 µg of mRNA per reaction using the High-Capacity cDNA Reverse Transcription kit (Applied Biosystems, Naerum, Denmark) according to the manufacturer's instructions. The reverse transcription was performed in a PTC-200 Thermal Cycler (MJ Research, Quebec, Canada).

The real-time quantitative PCR analysis was performed using a Lightcycler®

96 instrument and the FastStart DNA Master SYBR Green I mixture (Roche, Basel, Switzerland). A reaction mixture consisting of cDNA, primers (1 µM of each forward and reverse primer), water (PCR-grade), and master mix was used. Primers were designed in NCBI and obtained from Thermo Fischer Scientific (Additional file 1: Table S1). Cycle conditions were as follows: pre-incubation at 95 °C for 600 s, followed by 45 cycles of 95 °C for 10 s, 55 °C for 10 s, and 72 °C for 20 s. The efficiencies of the primer pairs were determined in-house by estimating the slope from a tenfold serial dilution calibration curve using the equation E = 10^(−1/slope)^. A set of three reference genes (*HPRT-1, YWHAZ,* and *SDHA*) was used to normalize the mRNA expression levels.

### Immunostaining

Cells were fixed in PBS with 3% (v/v) paraformaldehyde for 10 min and permeabilized in PBS with 0.1% (v/v) Triton x-100 for 5 min at room temperature. The samples were then blocked in PBS supplemented with 2% (w/v) bovine serum albumin (BSA) for 30 min at room temperature and incubated overnight at 4 °C with one of the antibodies listed in Additional file 1: Table S2. Following three washing steps with PBS + 2% (w/v) BSA for 5 min each, the samples were incubated for 30 min at room temperature with Alexa 488-conjugated secondary antibody (diluted 1:200), goat anti-rabbit or anti-mouse IgG (Molecular Probes, Leiden, the Netherlands), or Alexa 488-conjugated phalloidin (6 μM stock solution diluted 1:600, Life Technologies, Rødovre, Denmark), combined with propidium iodide (1.5 µM, Molecular Probes, Leiden, the Netherlands) for visualization of cell nuclei. Subsequently, the samples were washed twice in ice-cold PBS and mounted on coverslips. The samples were visualized under a confocal laser scanning microscope (Zeiss LSM 510, Carl Zeiss, Jena, Germany).

### Western blot analysis

For western blot analysis, endothelial cell monolayers were washed once with ice-cold PBS and the permeable supports removed from the filter inserts using a scalpel. Cells were lysed in cell extraction buffer (FNN0011, Thermo Fisher Scientific) supplemented with 1 mM phenylmethylsulfonyl fluoride, 1 mg/mL pepstatin, and complete protease inhibitor cocktail (04693116001, Roche) for 30 min on ice. The samples were then centrifuged at 14,000 g for 10 min at 4 °C. The supernatants were transferred to new Eppendorf tubes and the protein concentrations determined using the Bicinchonic Acid Kit according to the manufacturer's instructions. Total cell lysates were mixed with 4 × Laemmli sample buffer with 10% (v/v) β-mercaptoethanol. Samples were heated at 95 °C for 5 min. The proteins within the samples were separated on 4–15% pre-cast polyacrylamide gradient gels and electrotransferred to PVDF membranes using a Trans-Blot Turbo transfer system (Bio-Rad, Hercules, CA, USA). The membranes were incubated for 1 h with blocking buffer (1 × TBS, 0.1% (v/v) Tween-20 and 5% (w/v) skim milk), followed by incubation at 4 °C overnight with primary antibodies against the protein of interest (Additional file 1: Table S2). The membranes were then washed three times in TBS with 0.1% (v/v) Tween-20 (TBS-T) buffer for 5 min each. The washed membranes were incubated with horseradish peroxidase-conjugated secondary antibodies for 1 h at room temperature (Additional file 1: Table S2). Following another three washes with TBS-T for 5 min, the membranes were visualized using Amersham ECL-Prime Western Blotting Detection Reagent (GE Healthcare, Little Chalfont, UK) in a FlurorchemQ image Station (Alpha Innotech, Santa Clara, CA, USA).

### Uptake studies with [^3^H]-glucose and [^3^H]-leucine

Uptake studies were performed six days after seeding on the pre-coated permeable filter supports. Cells were washed twice and left to equilibrate for 15 min in glucose-free transport buffer, followed by the addition of 20 nM (1 µCi/mL) [^3^H]-glucose (49.5 Ci/mmol, PerkinElmer, Waltham, MA, USA) with or without 2 mM unlabeled glucose as an inhibitor, or in transport buffer, followed by the addition of 9.3 nM (1 µCi/mL) [^3^H]-leucine (107.6 Ci/mmol, PerkinElmer, Waltham, MA, USA) with or without 100 µM 2-aminobicyclo-(2,2,1)-heptane-2-carboxylic acid (BCH) as an inhibitor. HBSS (pH 7.4) supplemented with 10 mM HEPES, 0.05% (w/v) BSA, 0.375% (v/v) sodium carbonate with or without glucose was used as the transport buffer. Uptake was investigated at 30 s and 1, 2, 5, and 15 min at 37 °C on a temperature-controlled shaking table with a circular rotation of 90 rpm (Unimax 2010 Shaker). The uptake was stopped by removing the transport buffer and washing the cells three times with ice-cold HBSS buffer. The permeable supports were cut from the filter inserts using a scalpel and transferred to scintillation vials containing 2 mL of Ultima Gold scintillation fluid (PerkinElmer). The radioactivity was counted in a Tri-Carb 2910 TR Liquid Scintillation Analyzer (Perkin Elmer, Waltham, USA).

### Transport studies

Transcellular transport studies were performed directly in the culture medium six days after seeding on the pre-coated permeable filter supports. The transport was initiated by spiking with [^3^H]-digoxin (29.8 Ci/mmol, PerkinElmer, Waltham, MA, USA) to a final concentration of 25 nM (1 µCi·mL^−1^) or [^14^C]-mannitol (58 nCi/mmol, Perkin Elmer) to a final concentration of 8.6 µM (0.5 µCi/mL). The cells were placed on a temperature-controlled shaking table at 37 °C with a circular rotation of 90 rpm (Unimax 2010 Shaker).

Aliquots were removed from the receiver wells (50 µL from the apical or 100 µL from the basolateral compartment) after 15, 30, 45, 60, 90, and 120 min. The volume of the withdrawn samples was replaced with pre-heated DMEM-TES (37 °C). Samples were transferred to scintillation vials, and 2 mL of Ultima Gold scintillation fluid was added. The radioactivity was counted in a Tri-Carb 2910 TR Liquid Scintillation Analyzer. Transport studies were performed in both the apical-to-basolateral (A-B) and basolateral-to-apical (B-A) direction. The functionality of P-gp in the cells was evaluated by co-application of the specific P-gp inhibitor zosuquidar (0.4 µM), which was added to the culture medium in both chambers 15 min prior to the addition of [^3^H]-digoxin.

### Data treatment and statistics

The TEER measurements were standardized by multiplying the measure by the cross-sectional area of the permeable filter supports to obtain TEER in Ω·cm^2^.

The data obtained from the transport experiments were plotted as the total accumulated amount per cm^2^ as a function of time. The apparent permeability, P_app,_ was calculated from the steady-state fluxes that were calculated as the slope of the straight lines of the plot of Q against time. Thus, P_app_ was calculated as:

1 $$P_{app} = \frac{{J_{ss} }}{{C_{donor} }} = \frac{{Q_{t} }}{{C_{donor} \cdot A}}$$

where J_ss_ is the steady-state flux, C_donor_ represents the initial donor concentration, Q is the accumulated amount, and A is the area of the permeable supports. The efflux ratio was calculated as the ratio of P_app, B-A_ to P_app, A-B._

Statistical analyses were performed in Graph Pad Prism version 8 (GraphPad Software, La Jolla, CA, USA) using two-tailed Student's t-test or one-way ANOVA, followed by Tukey's multiple comparison test. Significance was set at P < 0.05. Experiments were performed on cells originating from at least three individual batches (n = 3) with at least three technical replicates (N = 3) for each experimental condition unless otherwise stated. Data are reported as mean ± standard deviation (SD).

## Results

### HIF-1α translocates to the nuclei of brain capillary endothelial cells cultured under hypoxic conditions

**Fig. 1:**
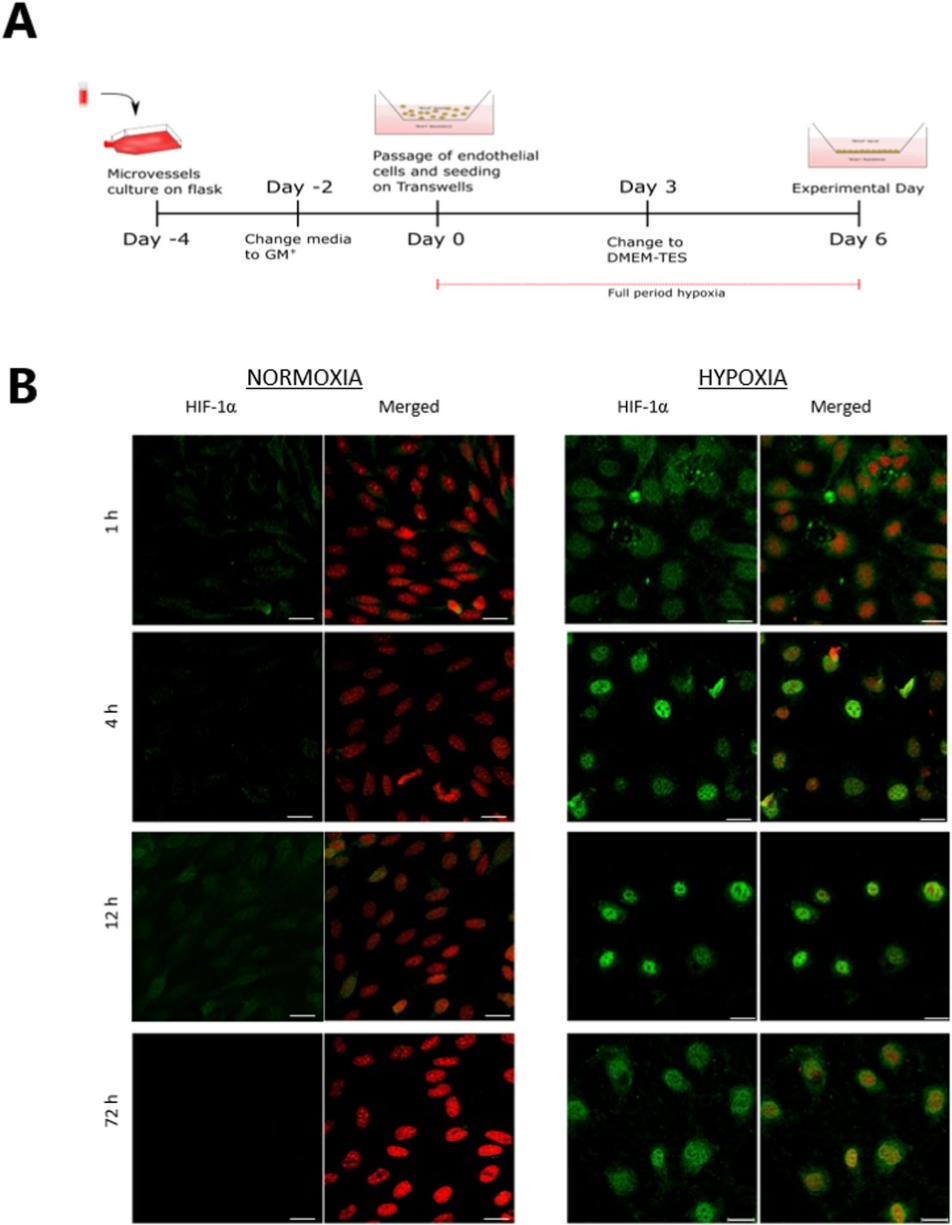
Chronic hypoxia induces stabilization and nuclear localization of HIF-1α in bovine brain capillary endothelial cells (BCECs). **A** Schematic overview of the timeline for the in vitro cultivation of BCECs in normoxic and hypoxic conditions. Bovine brain microvessels were seeded onto T75 flasks on day -4 and cultivated for five days. At day 0, outgrown BCECs were passaged and seeded on permeable filter supports. The cells were cultured in cell medium (GM-) for three days in a monoculture configuration under normoxic or hypoxic conditions. The culture medium was changed to DM-TES medium on day 3, and the cells were ready for experiments on day 6. **B** Cell monolayers were immunostained for HIF-1α (green) after 1, 4, 12, and 72 h upon changing the medium on day 3. The samples were counterstained with propidium iodide (red) to visualize cell nuclei. Single and merged images are presented as XY frontal focal planes. Scale bar = 20 μm. Images are representative of three individual experiments in triplicate (n = 3, total N = 9)

The effects of hypoxia on cultured brain endothelial cells were investigated by establishing BCEC monoculture under normoxic and hypoxic conditions (Fig. 1A). The experimental setup was designed to mimic the oxygen tension that brain endothelial cells are exposed to during the development of the CNS vasculature [19, 20]. First, we examined whether chronic exposure of BCECs to hypoxia (1% O_2_) affects the stability of HIF-1α by investigating the time-dependent changes (1, 4, 12, and 72 h after changing to differentiation medium) in HIF-1α localization (Fig. 1B). The expression of HIF-1α was negligible in cells cultured under normoxic conditions. Immunostaining in cells cultured under hypoxic conditions revealed "punctured" structures in the cytoplasm at 1 h, indicative of stabilization of the protein. HIF-1α translocated and concentrated in the cell nuclei by 4 h (Fig. 1B) and exhibited an exclusively intranuclear distribution pattern at 12 h in hypoxic cells. At 24 h, HIF-1α gradually delocalized from the cell nuclei and redistributed in the cytoplasm. However, some nucleoplasmic localization was retained up to the experimental day (Day 6/72 h after change to differentiation medium) in the cells exposed to hypoxia. Thus, the BCECs were capable of responding to hypoxia.

### Hypoxia induced important phenotypic BBB traits and did not affect the barrier integrity in bovine brain endothelial cell monolayers

**Fig. 2:**
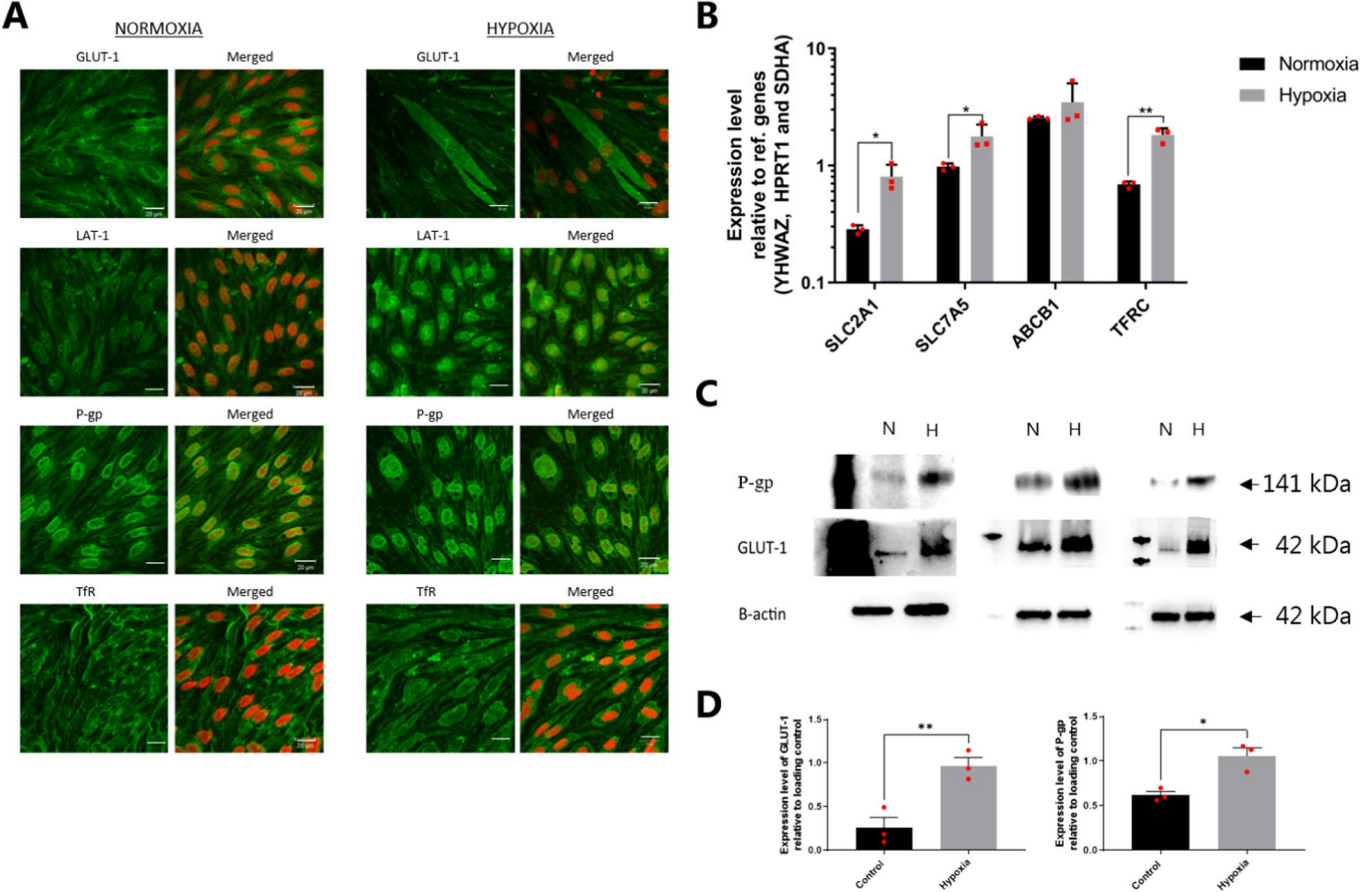
The gene and protein expression and localization of selected transporter proteins in bovine endothelial cells cultured in normoxic and hypoxic conditions. **A** Immunostaining of GLUT-1, LAT-1, P-gp, and TfR (each in green) in bovine brain capillary endothelial cells (BCECs) on Day 6 upon culture in normoxic and hypoxic conditions. Propidium iodide is red. Single or merged images are presented as XY frontal focal planes. Scale bars = 20 μm. Images are representative of three individual experiments in triplicate (n = 3, total N = 9). **B** Transcript levels of SLC2A1 (GLUT-1), SLC7A5 (LAT-1), ABCB1 (P-gp), and TFRC (TfR) in normoxic and hypoxic BCECs were analyzed by RT-qPCR. Expression levels were normalized to YHWAZ, SDHA, and HPRT-1. Data are shown as mean ± SD of three individual batches of triplicates (n = 3, total N = 9). **C** Immunoblots of GLUT-1 and P-gp in cells cultured under normoxic and hypoxic conditions, and β-actin as loading control. The left lane represents the protein ladder. D) Quantification of GLUT-1 (left) and P-gp (right) protein levels relative to β-actin. Data are representative of three individual experiments (n = 3). The mean values were compared by Student's t-test; *p < 0.05, **p < 0.005

Expression of selected BBB transport systems (GLUT-1, LAT-1, P-gp, and TfR) was investigated by immunocytochemistry, Western blotting and qPCR to determine whether BCECs cultured under hypoxic conditions recapitulate the in vivo features of the brain capillary endothelium (Fig. 2).

GLUT-1 appeared to mainly localize in the cytosol in cells maintained under normoxic conditions (Fig. 2A). In contrast, GLUT-1 was more localized at the cell membrane, with heterogeneous distribution in cells maintained under hypoxic conditions. Western blotting of cell lysates revealed that the expression levels of GLUT-1 and P-gp were significantly increased (p = 0.01). GLUT-1 showed approximately threefold in hypoxic cells relative to normoxic cells, while the increase in P-gp expression was 1.7 fold (Fig. 2C). Immunostaining of LAT-1 revealed predominant perinuclear staining with minor staining at the cell borders in cells exposed to chronic hypoxia, whereas LAT-1 appeared to be more diffuse in the cytosol and cell borders in cells cultured under normoxic conditions (Fig. 2A). For P-gp, a cytosolic and perinuclear expression pattern was observed under both culture conditions. TfR staining indicated expression throughout the cells, with a more predominant presence at the cell membrane in both groups. However, higher vesicle density was present in the hypoxic cells, as indicated by the punctured structures, which were not observed to the same degree in normoxic cells. The mRNA expression levels of SLC2A1 (~ threefold, p = 0.014), SLC7A5 (~ twofold, p = 0.039), and TFRC (~ threefold, p = 0.0016) were significantly higher in the hypoxic cells than the normoxic cells. The expression level of ABCB1 was also increased, though not significantly.

**Fig. 3:**
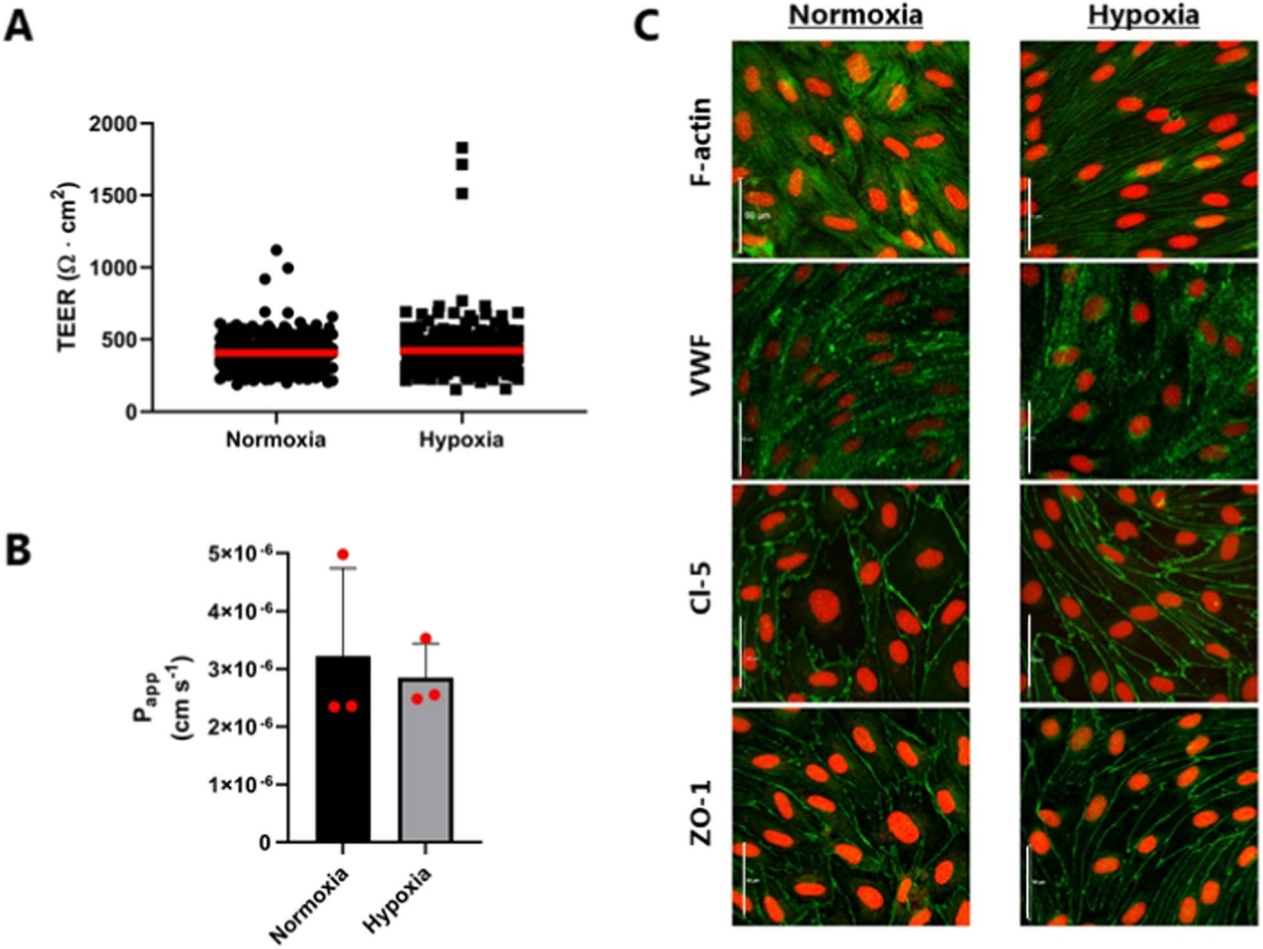
Transendothelial electrical resistance (TEER) measurements, mannitol permeability and immunocytochemical characterization of normoxic and hypoxic bovine brain capillary endothelial cells (BCECs). **A** TEER was measured across the normoxic and hypoxic cell monolayers on the day of the experiment (day 6). Red bold lines show the mean values. Measurements were normalized to the area of the filter supports (total N = 230). **B** The apical-to-basolateral permeability of [14C]-Mannitol was examined over a time course of 120 min. P_app_ values were calculated from the steady-state fluxes and determined over 120 min., and calculated from steady state fluxes. Data are shown as mean ± SD of three individual batches of three individual permeable supports (n = 3, total N = 9). *p < 0.05. **C** Immunocytochemical characterization of normoxic and hypoxic BCEC monolayer on day 6. The cell monolayers were immunostained for filamentous actin (F-actin), von Willebrand factor (VWF), claudin-5 (Cl-5), and zonula occludens-1 (ZO-1). All samples were counterstained with propidium iodide (red) to visualize cell nuclei. Scale bars = 50 μm. Images are representative of three individual experiments in triplicate (n = 3, total N = 9)

Next, we investigated the effects of chronic hypoxic exposure on functional tightness and barrier integrity by measuring TEER, transendothelial fluxes of the paracellular transport marker, [^14^C]-mannitol, and examining the cell morphology and TJ protein localization using immunocytochemistry (Fig. 3).

The mean electrical resistance across the cells cultured under hypoxic conditions was 421 ± 187 Ω∙cm^2^, which is comparable to normoxic cells (405 ± 129 Ω∙cm^2^). For several batches, a higher TEER was observed in cells cultured under hypoxic conditions. The apparent permeability of [^14^C]-mannitol was estimated to 3.2 ± 1.5 × 10^–6^ cm s^−1^ across normoxic cells, and not affected by the pro-longed hypoxic cultivation (2.8 ± 0.6 × 10^–6^ cm s^−1^).

The BCECs exhibited F-actin enrichment at cell contacts and exhibited a spindle shape with thin and elongated cells in both culture conditions. Thus, no morphological changes were observed. The endothelial-specific Von Willebrand factor was expressed throughout the cells in both conditions. The TJ proteins Cl-5 and ZO-1 demonstrated continuous expression at the cell borders with no differences between normoxic and hypoxic cell monolayers. Based on these results, culturing under hypoxic conditions did not induce any changes in the BBB TJs, demonstrating the ability of the brain endothelial cells to develop and maintain a tight barrier under hypoxic conditions.

**Fig. 4:**
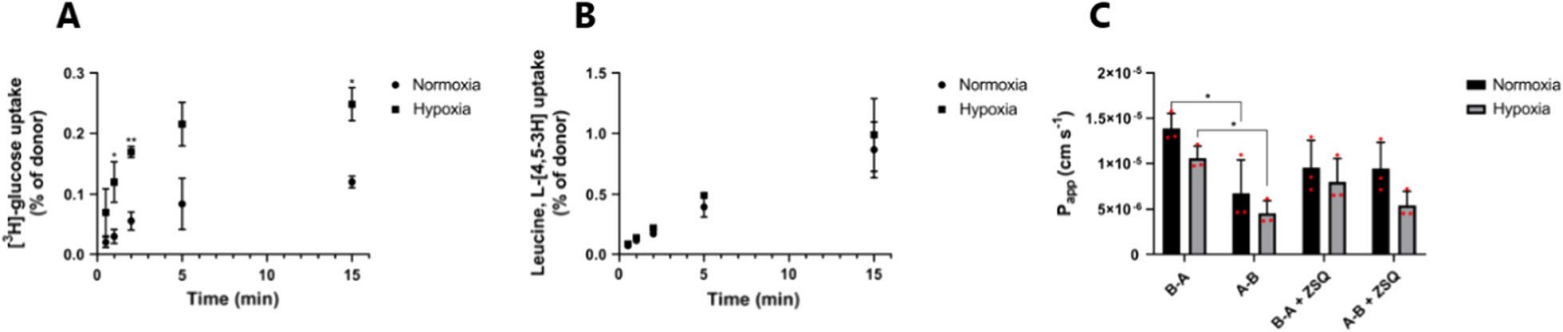
The activities of GLUT-1, LAT-1, and P-gp in bovine brain capillary endothelial cells (BCECs) differentiated under normoxic (circles and black bars) and hypoxic (squares and grey bars) conditions. **A** Luminal uptake of [3H]-glucose and B) L-[4,5-3H]-leucine into BCECs was examined as a function of time**.** The donor concentrations of [3H]-glucose and L-[4,5-3H]-leucine was 20 and 9.5 nM, respectively. The uptake data are shown as mean ± SD of three individual cell batches of duplicates (n = 3, total N = 6). **C** Bidirectional transport of [3H]-digoxin across BCEC monolayers in the absence and presence of 0.4 μM zosuquidar (ZSQ). P_app_ values were calculated from the steady-state fluxes and determined over 120 min. The donor concentration of [3H]-digoxin was 25 nM. B-A represents transport from the basolateral to apical direction, while **A**–**B** incidates transport from apical to basolateral. Data are shown as mean ± SD of three individual batches of three individual permeable supports (n = 3, total N = 9). *p < 0.05

We measured the time-dependent luminal uptake of [^3^H]-glucose and [^3^H]-leucine to assess the transport capacity of GLUT-1 and LAT-1 (Fig. 4A and B).

The time-dependent uptake of [^3^H]-glucose was linear for 2 min, followed by an apparent plateau under both conditions. However, the plateau under hypoxic conditions was markedly higher than the plateau in normoxic cells, which corresponded to the higher expression of GLUT-1. To confirm that the increased uptake of [^3^H]-glucose was primarily caused by increased activity of the Glut-type transporter, we examined the uptake of [^3^H]-glucose for 1 min in glucose-free uptake buffer, and in sodium- and glucose-free buffer (Additional file 1: Fig. S1). The uptake of [^3^H]-glucose was not diminished in the absence of sodium, but it was inhibited by the inclusion of non-labeled glucose in both the sodium-free uptake buffer and the buffer with sodium. This indicates that the uptake of glucose was carrier-mediated and sodium-independent, suggesting that the increased cellular uptake of [^3^H]-glucose into hypoxic cells was mediated mainly by a Glut-type transporter. The time-dependent uptake of [^3^H]-leucine remained linear for at least 15 min in both groups, and no differences were observed between the groups (Fig. 4B). The uptake of [^3^H]-leucine was diminished by the addition of 2-aminobicyclo-(2,2,1)-heptane-2-carboxylic acid (BCH; Additional file 1: Fig. S1), indicating that LAT-1 mediated the leucine uptake. Bidirectional transport of the prototypical P-gp substrate [^3^H]-digoxin in the absence and presence of the P-gp inhibitor zosuquidar was investigated to assess the functional expression of P-gp in cells cultured under hypoxic conditions (Fig. 4C). [^3^H]-digoxin had significantly higher P_app, B-A_ than P_app, A-B_ (6.8 ± 3.6 × 10^–6^ cm s^−1^ vs 14 ± 1.7 × 10^–6^ cm s^−1^, p = 0.036), with a resulting efflux ratio of 2.1 ± 0.4 across normoxic cell monolayers. Similarly, [^3^H]-digoxin exhibited a significantly higher P_app, B-A_ as compared to P_app, A-B_ (10.6 ± 1.3 × 10^–6^ cm s^−1^ vs 4.6 ± 1.4 × 10^–6^ cm s^−1^, p = 0.0058) in cells cultured under hypoxic conditions.

The efflux ratio was calculated to 2.4 ± 0.2, which was slightly higher than the ratio in normoxic cells, albeit non-significant. Moreover, no significant differences were observed when comparing the P_app,A-B_ and P_app,B-A_ values from hypoxic cells against their counterparts in normoxic cells cells. Co-administration of the P-gp inhibitor, zosuquidar, increased the transport from the basolateral to apical direction, while a decrease was observed in the transport from apical to basolateral direction in both cell groups. Zosuquidar diminished the efflux ratios to 1.0 ± 0.1 and 1.5 ± 0.1 in normoxic and hypoxic cells, respectively, indicating functional P-gp in both cell groups.

### DFO-mediated HIF-1α stabilization under normoxic conditions tightened the endothelial junctions

**Fig. 5:**
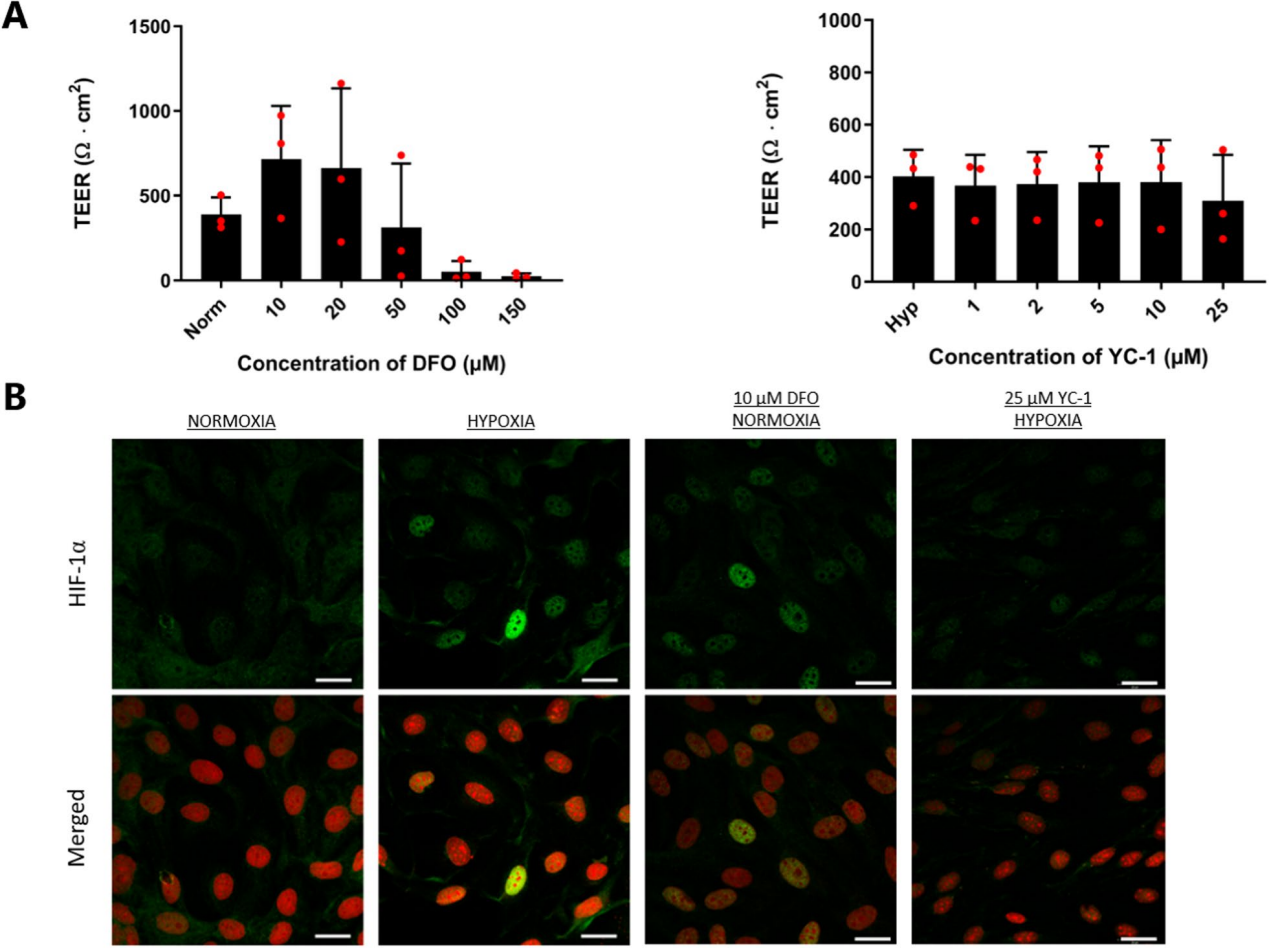
Effects of DFO and YC-1 on the transendothelial electrical resistance (TEER) measurements and localization of HIF-1α. **A** TEER was measured across the cell monolayers cultured in the absence and presence of various concentrations of DFO or YC-1 under normoxic and hypoxic conditions on the experimental day. Measurements were normalized to the area of the filter supports. Data are shown as mean ± SD of three individual batches of two individual permeable supports (n = 3, total N = 6). **B** Cell monolayers were immunostained for HIF-1a (green) on day 6 of culture on Transwells and were counterstained with propidium iodide (red) to visualize cell nuclei. Cells were cultured under normoxic conditions without and with DFO, and under hypoxic concentrations without and with YC-1. The concentrations of DFO and YC-1 were 10 and 25 μM, respectively. Scale bar = 20 μm. Images are representative of three individual experiments in triplicate

We supplemented the culture medium with DFO to chemically stabilize HIF-1α under normoxic conditions and with YC-1 to destabilize HIF-1α in cells cultured under hypoxic conditions throughout the culture period on permeable supports to investigate whether the observed effects were HIF-1α-dependent. The barrier tightness of cells cultured in the presence of increasing concentrations of DFO (10–150 µM) or YC-1 (1–25 µM) was examined by measuring the TEER across the cell monolayers on Day 6 to examine the dose-dependent effect of DFO and YC-1 on the barrier integrity (Fig. 5A). Under control conditions, the mean TEER was 389 ± 100 Ω cm^2^. TEER increased at 10 and 20 µM DFO to 715 ± 313 Ω cm^2^ and 663 ± 471 Ω cm^2^, respectively. TEER was apparently unaffected at 50 µM DFO, whereas 100 µM and 150 µM DFO caused complete barrier breakdown. Destabilization of HIF-1α with increasing concentrations of YC-1 under hypoxic conditions did not affect the barrier integrity. The TEER was in the range of 368–403 Ω cm^2^ when YC-1 was applied in concentrations up to 10 µM, whereas 25 µM YC-1 caused a slight, but not significant, decrease to 308 ± 176 Ω∙cm^2^. Based on these dose-dependent effects of DFO and YC-1 on the barrier integrity, we chose to apply 10 µM DFO to stabilize HIF-1α under normoxic condition and 10 µM YC-1 to destabilize HIFs under hypoxic condition. We next investigated the stability and localization of HIF-1α in BCECs cultured under normoxic and hypoxic conditions without and with 10 µM DFO and 10 µM YC-1, respectively (Fig. 5B). We found that 10 µM DFO was sufficient to induce HIF-1α translocation to the nucleus under normoxic conditions, whereas 10 µM YC-1 cultivation under hypoxia suppressed this translocation.

**Fig. 6:**
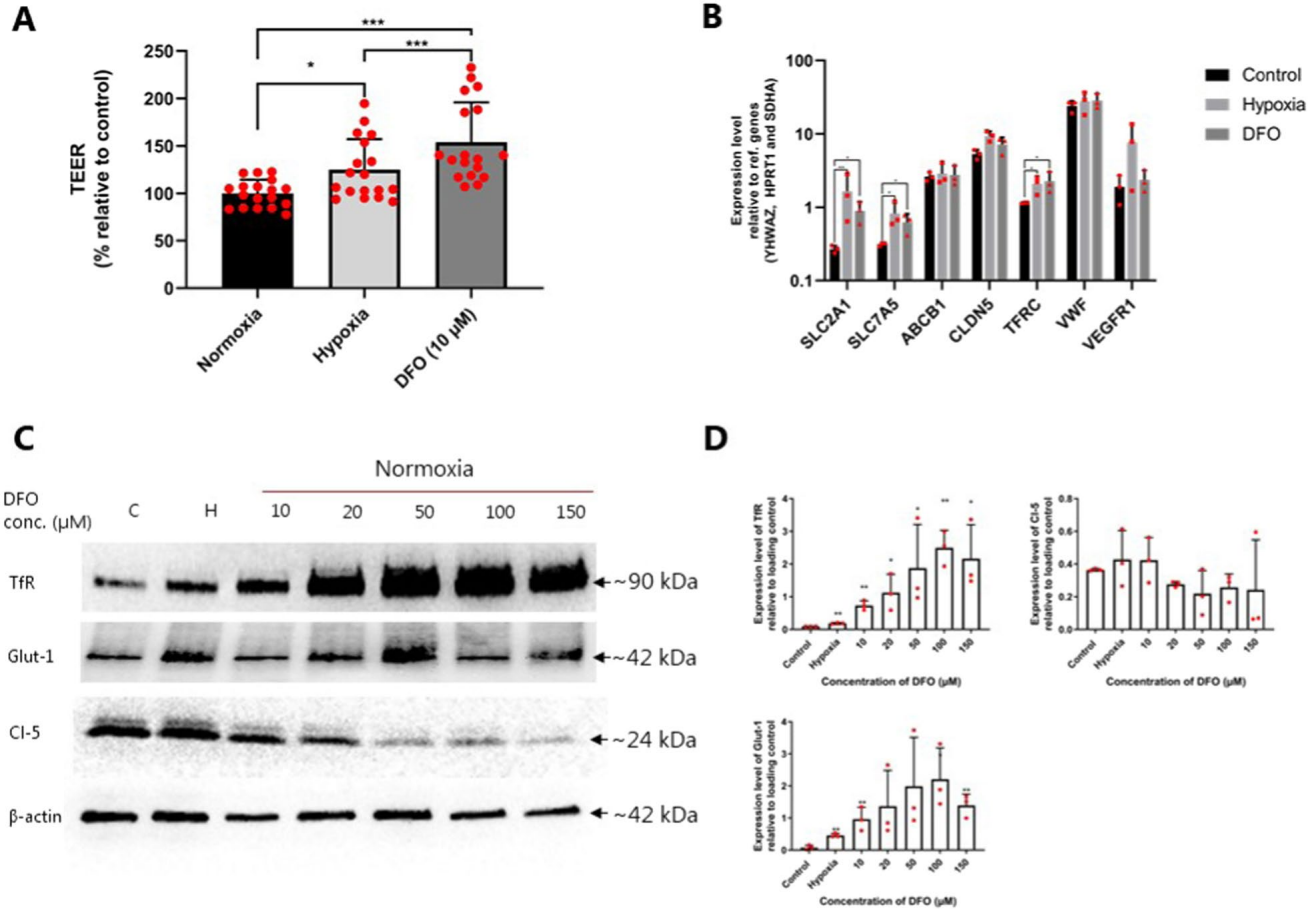
Transendothelial electrical resistance (TEER) measurements and localization of HIF-1α in cells cultured in the absence and presence of 10 μM DFO under normoxic conditions or hypoxic conditions. **A** TEER was measured across the cell monolayers after six days of culture on permeable filter supports (i.e., experimental day). TEER is plotted as % of relative to normoxic cells without DFO (control; n = 3, total N = 18). **B** The mRNA expression level of SLC2A1, SLC7A5, ABCB1, CLDN5, TFRC, and VEGFR1 under normoxic and hypoxic BCECs, and cells cultured with 10 μM DFO under normoxic conditions. Expression levels were normalized relative to YHWAZ, SDHA, and HPRT-1. Data are shown as mean ± SD of three individual batches of triplicates (n = 3, total N = 9). **C** Immunoblots of TfR, GLUT-1, and Cl-5. D) Quantification of TfR and Cl-5 protein levels relative to the loading control β-actin. Data are representative of three individual batches (n = 3)

We evaluated the TEER and transcript levels of SLC2A1, SLC7A5. ABCB1, CLDN5, TFRC, VWF, and VEGFR1 (Fig. 6A and B). Similar to hypoxic conditions, induction of HIF-1α in BCECs with 10 µM DFO treatment under normoxic conditions resulted in a significant increase in the expression of SLC2A1 (~ threefold, p = 0.022), SLC7A5 (~ twofold, p = 0.046), and TFRC (~ twofold, p = 0.044). The CLDN5 transcript levels revealed a tendency to increase in BCECs cultured under hypoxic conditions and in the presence of DFO under normoxic conditions. The VEGFR1 mRNA levels revealed non-significant upregulation in cells cultured under hypoxic conditions compared to normoxic cells cultured in the absence or presence of 10 µM DFO.

**Fig. 7:**
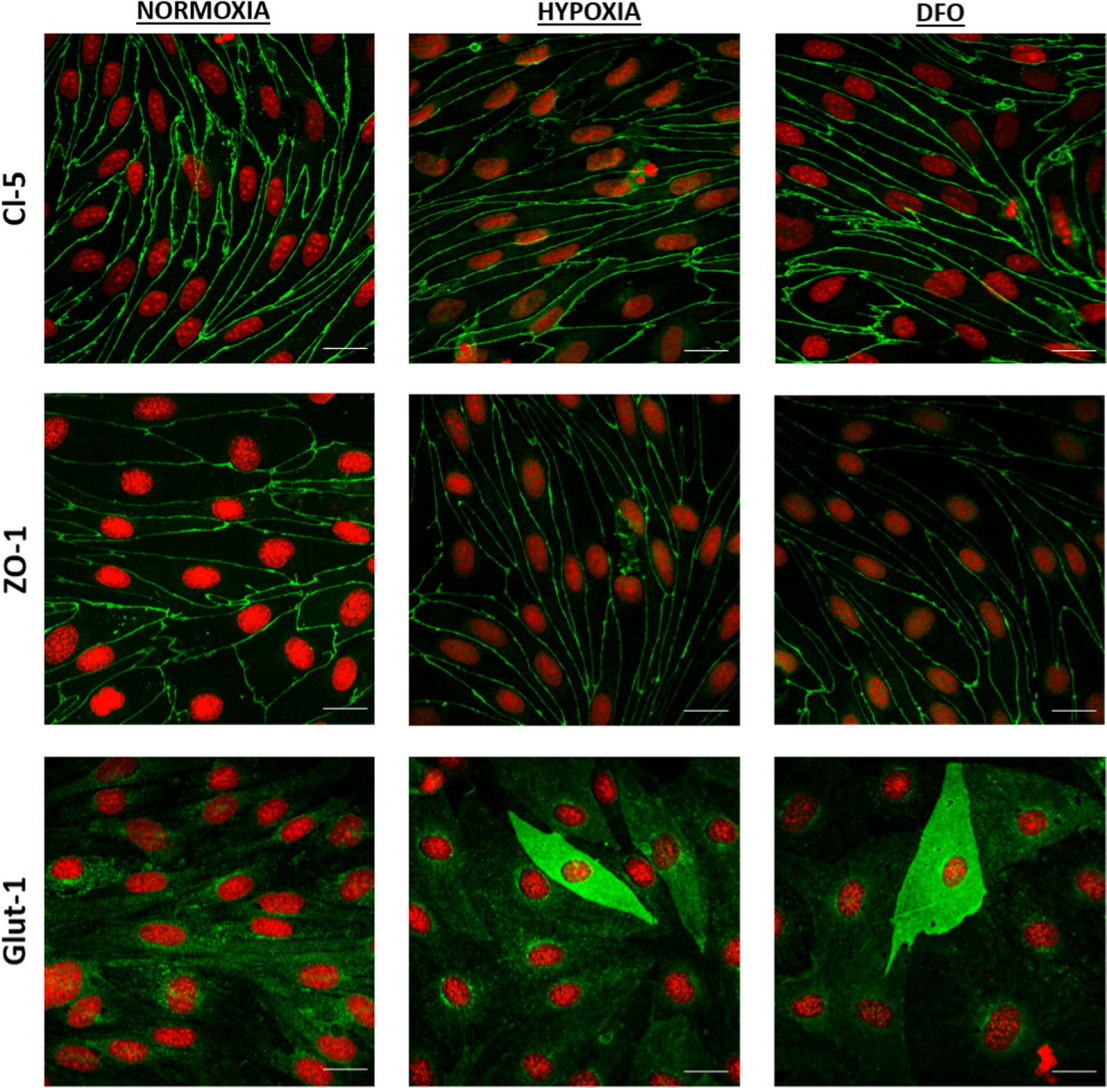
Immunocytochemical characterization of BCEC monolayers cultured under normoxic conditions in the absence or presence of 10 μM DFO and under hypoxic conditions. The cell monolayers were immunostained for filamentous claudin-5 (Cl-5), zonula occludens-1 (ZO-1), and GLUT-1. All samples were counterstained with propidium iodide (red) to visualize cell nuclei. Scale bars = 20 μm. Images are representative of three individual experiments in triplicate

The GLUT-1 and TfR protein expression levels significantly increased in hypoxic BCECs compared to normoxic cells (GLUT-1: p = 0.0065 and TfR: p = 0.0039), whereas DFO treatment induced a more pronounced increase than hypoxia (Fig. 6C, D). No differences were observed in Cl-5 protein levels in hypoxic BCECs or cells cultured under normoxic conditions in the absence or presence of DFO at concentrations up to 10 µM (Fig. 6C, D). Similar to hypoxia, normoxic HIF-1α stabilization with 10 µM DFO did not induce morphological changes or changes in TJ localization (Fig. 7).

### Hypoxia induces barrier tightening of confluent brain endothelial cell monolayers

Lastly, the effects of hypoxia were assessed on confluent brain capillary endothelial cells.

For this purpose, we performed a new set of experiments, where cells were allowed to grow into a confluent monolayer until Day 6/Experimental Day prior to exposure to hypoxia (1% O_2_) for 48 h. The time-dependent changes in barrier integrity of the cell monolayers were determined by monitoring the TEER.

**Fig. 8:**
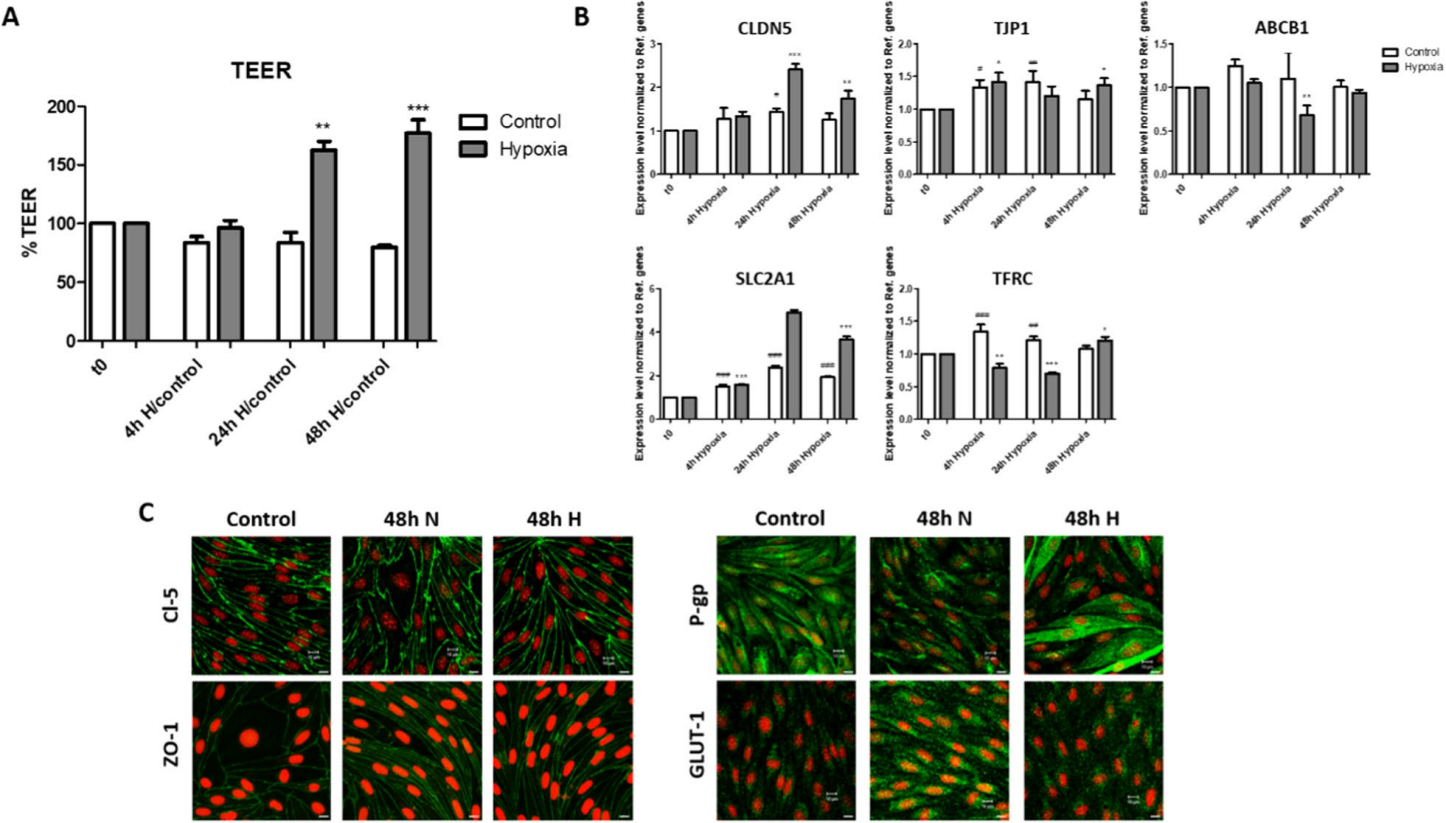
Transendothelial electrical resistance (TEER) measurements, mannitol permeability and immunocytochemical characterization of confluent brain capillary endothelial cells (BCECs) exposed to hypoxia. **A** TEER was measured across cell monolayers from the Experimental Day (day 6), where cells were exposed to hypoxia (grey bar) or normoxia/control (white bars) and for 48 h. Measurements were normalized to normoxic control (0 h time point). Data are shown as mean ± SD of three individual batches of triplicates (n = 3, N = 3). **B** The mRNA expression level of CLDN5, TJP1 (ZO-1), SLC2A1 (GLUT-1), TFRC and ABCB1 (P-gp) in confluent BCECs exposed to hypoxia (grey bars) or normoxia (white bars). Expression levels were normalized relative to normoxic control at time point 0 h. YHWAZ, SDHA, and HPRT-1 were used as reference genes. Data are shown as mean ± SD of three individual batches of triplicates (n = 3, N = 3). **C** Immunocytochemical characterization of confluent BCEC monolayer exposed to hypoxia or normoxia for 48 h. Control indicates cells at time point 0 h. The cell monolayers were immunostained for claudin-5 (Cl-5), zonula occludens-1 (ZO-1), P-glycoprotein (P-gp) and GLUT-1. All samples were counterstained with propidium iodide (red) to visualize cell nuclei. Scale bars = 10 μm. Images are representative of three individual experiments in triplicate (n = 3, N = 3)

Interestingly, we noted a significant increase (> 50%) in the electrical resistance after 24 h in confluent cell monolayers exposed to hypoxia. This markedly increase remained stable for at least up to 48 h, indicating a hypoxia-mediated induction of barrier tightness. The TEER across cell monolayers that were kept under normoxic conditions remained stable during the 48 h. We measured the transcript levels of CLDN5 and TJP1 (ZO-1) to further confirm the TEER read outs, and also SLC2A1, ABCB1 and TFRC were included (Fig. 8B). CLDN5 and SLC2A1 transcript levels peaked in both treatments at 24 h and subsequently declined. Significantly higher CLDN5 and SLC2A1 levels were found in confluent cell monolayers that were exposed to hypoxia. For TJP1, transcript levels were found to be significantly higher at 4 h in both groups and remained stable for up to 48 h. TFRC level underwent a significant decrease in cells exposed to hypoxia at 4 and 24 h, but increased to significantly higher level at 48 h as compared to control. TFRC were found to be upregulated in normoxic cells at 4 and 24 h, and declined to baseline level at 48 h. These hypoxia-exposed cells also formed well developed tight junctions containing ZO-1 and Cl-5, and GLUT-1 and P-gp transporters at their membranes (Fig. 8C).

## Discussion

### Endothelial cell monolayer integrity was maintained under hypoxia, while GLUT-1, LAT-1, P-gp and TfR expression increased

Several studies have indicated that hypoxic conditions may be detrimental to brain capillary endothelium integrity via, for example, TJ opening [6, 21–25]. However, the effects of hypoxia on BCECs are often investigated after cells have grown into confluency and formed a functional barrier. In the present study, we aimed at investigating whether hypoxia had effects on the paracellular integrity and the expression of selected BBB phenotype markers by culturing BCECs under hypoxic conditions upon seeding on permeable support for six days to mimic the hypoxic environment that BCECs are exposed to during early vascularization [19, 20]. We found that the barrier integrity was maintained under chronic hypoxic culture concomitant with increased expression of markers of a mature BBB phenotype. Chemical induction of a hypoxic response using DFO to activate the HIF-1α signaling pathway mimicked the hypoxic response, and DFO in moderate concentrations did even increased the tightness relative to the untreated controls. Our findings agree with studies on brain vascularization that have demonstrated that BCECs form mature TJ zones in the low-oxygen environments present in the embryonic brain parenchyma during early vascularization (as reviewed by Saunders et al. [14]). Ek et al. demonstrated that the adjacent endothelial cells form a functionally effective barrier mechanism towards small molecules in the very early stages of brain vascularization [13]. This suggests the intriguing possibility that hypoxia may also regulate barrier-genesis in addition to angiogenesis, leading to the hypothesis that BCECs can develop TJ complexes under hypoxic conditions. This is supported by expression studies showing that Cl-5 is present and properly localized in brain capillaries in mice [2] and human fetuses [26]. In vivo studies on horseradish peroxidase permeation across the BBB demonstrated some brain uptake [27–29], but this could be explained by an increased transcellular passage rather than a paracellular leak [12], which is not down-regulated in developing brain vasculature before pericyte recruitment [12]. Our findings indicate that a hypoxic environment is certainly not a hindrance to the generation of an effective junctional alignment in BCECs or for the expression of some important BBB markers. This was also supported by the results observed when allowing cells to grow into confluency followed by a 48 h hypoxic-exposure, where the barrier tightness was found to be enhanced after 24 h and 48 h as compared to control cells that were kept under normoxic conditions. This increase in TEER translated into higher transcript levels of CLDN5. Thus, hypoxia may not necessarily be detrimental to the paracellular integrity or to the BBB phenotype of BBB. We instead observed an increase in TfR, GLUT-1 and LAT-1 that potentially could lead to a higher transcellular passage, as seen in the study on the developing embryonic brain vasculature [12].

GLUT-1 upregulation through HIF-1 signaling is a well-known consequence of hypoxia in multiple cell types, including lung epithelial cells, cancer cells, and BBB endothelial cells [30–33]. In this study, we observed GLUT-1 upregulation at the mRNA, protein, and functional levels. Furthermore, TfR up-regulation occurs in response to hypoxia, and it is coupled directly with HIF-1 signaling [34]. LAT-1 was also found to be upregulated by both hypoxia and DFO treatment as judged by the mRNA expression. However, this upregulation did not translate into higher uptake of [^3^H]-leucine or function of LAT-1, presumably due to limited translocation to the cell membranes or lack of translation to protein. Moreover, P-gp was found the be upregulated at protein level in cells cultured under hypoxic conditions, and bidirectional transport study using the well-characterized P-gp substrate, [^3^H]-digoxin, to probe the activity of P-gp, revealed that the substrate exhibited a slightly higher efflux ratio, albeit non-significant, in hypoxic cells.

Together with the maintenance of TJ integrity, the up-regulation of known BBB phenotype markers indicates that chronic hypoxia may induce a more BBB-like phenotype in cultured BCECs as previously reported by Park et al. in a study on brain endothelial cells derived from human induced pluripotent stem cells [35].

In contrast to our results, Boado et al. reported a downregulation of SLC7A5 (LAT-1) transcripts in bovine BCECs exposed to hypoxic conditions for 24 h after growing cells to 90% confluence; the level subsequently returned to that of normoxic cells after 48 h of hypoxia [36]. However, hypoxia-induced up-regulation of *SLC7A5* has been reported in other cell types [31, 37].

The low oxygen tension could interact simultaneously with Wnt signaling pathways and HIF-1 signaling to mediate BBB differentiation [38–41]. The interplay between HIF-1α and Wnt/β-catenin signaling is essential for early brain vascularization. Western blot analysis revealed that β-catenin is upregulated by culturing endothelial cells under chronic hypoxia (Additional file 1: Fig. S2). Immunostaining revealed that β-catenin was mainly localized at the junctional zones and did not translocate to the nuclei (Additional file 1: Fig. S2). It has previously been seen that β-catenin accumulation at the junctional zones could promote stability of the TJ proteins [42]. It is likely that the effects of chronic hypoxia in the present study were mainly due to hypoxia-induced HIF-1α signaling.

### Oxygen–glucose deprivation versus hypoxia

The effects of hypoxia are often evaluated in concert with glucose deprivation in vitro to mimic the effects of ischemic stroke on the BBB. This can result in markedly different responses in the endothelial cells than hypoxia alone, especially as endothelial cells rely mainly on anaerobic glycolysis for ATP production [43]. Few studies have attempted to distinguish between hypoxia and glucose deprivation [21, 44, 45]. A number of studies have demonstrated increased BBB permeability as an important factor after ischemic stroke [46–48]. The insult has also been verified in several in vitro studies with cultured endothelial cells using oxygen–glucose deprivation (OGD) treatment after cells have grown to confluency and formed a functional barrier [21, 47, 49, 50]. However, many of the observed effects of OGD in a BBB in vitro model were recently demonstrated to be similar to those induced by a simple culture medium change [51]. This makes it difficult to ascribe the induced barrier leak directly to the hypoxic/aglycemic conditions in in vitro studies [51].

Contrarily, contradictory results have been reported regarding BBB breakdown in studies of hypoxia alone. Hypoxia has been shown to disrupt membrane localization of Cl-5 and ZO-1 in brain capillary endothelial cells in vitro (1–5% of O_2_), resulting in barrier breaches and enhanced paracellular transport to various tracers [6–8]. However, all these in vitro studies are performed after cells have grown to confluency. Brain capillaries from mice subjected to hypoxia do not show any differences in the expression of CLDN5, ZO-1, or OCLN at the mRNA level, and no considerable alterations were observed recently in the TJ strand network [46]. Furthermore, Li et al. observed that the BBB integrity was maintained in mice exposed to mild hypoxic conditions, and the BCECs demonstrated induced expression of ZO-1 and Cl-5 [52]. We also demonstrated that the hypoxia-exposure to confluent cell monolayer resulted in barrier tightening. Similar results were observed in human pulmonary microvascular endothelial cells grown to confluence under hypoxic conditions, which exhibited tighter and less permeable monolayers than cells grown under normoxic conditions [53]. On the other hand, Schoch et al. have previously demonstrated that exposing mice to hypoxic conditions (6–12% oxygen) results in vascular leakage in brain capillaries [22]. It should, however, be noted that alternation of TJ protein expression is often seen as a result of BBB injury. Therefore, it is still unclear which effects, if any, hypoxia has on TJ integrity, though our study and other studies clearly indicate that it does not cause any disruption in the TJs.

### DFO induces translocation of HIF-1α to the nucleus and increases the TEER

We observed that HIF-1α stabilization with lower concentrations of DFO (10 and 20 µM) under normoxic conditions resulted in a marked increase in the TEER compared to normoxic cells, whereas the higher concentrations of DFO (50–150 µM) resulted in a compromised barrier. This novel finding is intriguing, as it was not observed in the cells exposed to hypoxia. Therefore, the significant increase and decrease in TEER upon cultivation with low and high concentrations of DFO, respectively, are likely independent of HIF-1α and is presumably caused by other cellular events that occur concurrently with HIF-1α stabilization as a consequence of iron chelation. DFO has previously demonstrated protective effects on BBB integrity in rats with subarachnoid and intracerebral hemorrhage [54, 55], presumably by reducing the iron overload. Iron is a potent generator of reactive oxygen species, which have been reported to disrupt TJ complexes[56]. However, HIF-1α signaling has also been reported to increase the expression of TJ proteins, and chemically stabilization of HIF-1α with dimethyloxalylglycine (a competitive inhibitor of prolyl hydroxylase domain enzymes) increases the expression of claudin-1 in esophageal epithelial cells in vivo and ex vivo in human biopsy tissues from the esophagus [57]. Thus, the effects by DFO could also be directly linked to HIF-1-α related signaling, possibly in combination with the iron-chelating effects. Taken together, the data suggest that a low to moderate concentration of DFO has protective and enhancing effects on barrier integrity and can be a promising therapeutic agent for the treatment of barrier breakdown in several diseases.

### Other cell types from the neurovascular unit may lead to additional responses to hypoxia

Cerebral astrocytes respond to hypoxia and HIF-1α signaling via increased synthesis and excretion of VEGF both in vitro and in vivo [58–60]. VEGF is a potent mediator of vascular permeability by promoting leakage and acts directly on endothelial cells through two high-affinity receptors, fms-like tyrosine kinase (VEGFR1, flt-1) and kinase domain region (VEGFR2, KDR). Treatment of cultured endothelial cells with VEGF induces hyperpermeability [61], which has also been shown in vivo upon stimulation from the brain compartment [62] 63]. Co-culturing astrocytes with bovine BCECs results in the barrier properties of the endothelium being affected to a higher degree than in monoculture when the endothelial cells were subjected to hypoxia alone [50]. Also, Li et al. previously showed that incubation of OGD-treated neurons in co-cultures of bEnd-3 cells and astrocytes induced barrier disruption by activating astrocytes to secrete VEGF [64]. This indicates that intercommunication between astrocytes and endothelial cells upon hypoxic insult may be an important factor for the increased vascular permeability seen in the referred studies. In contrast, pericytes and astrocytes have been reported to exhibit barrier protective effects on cultured BCECs exposed to hypoxia [24, 65, 66], even though pericytes also are reported to release angiogenic molecules, such as VEGF, in response to hypoxia exposure [67, 68]. Earlier studies suggested that neurons are essential for regulating the CNS vasculature density, as neurons have high energy turnover and demand a continuous oxygen supply. The absence of these neurovascular unit cells in our study may have affected the endothelial responses to hypoxia. Thus, the correlation of results from hypoxia studies in in vitro models of BCECs to the in vivo situation may be challenging.

## Conclusions and perspectives

The findings in the present study indicate that the culture of endothelial cells under hypoxic conditions led to upregulation of the specific BBB transporter systems, P-gp, GLUT-1, LAT-1 (at mRNA level), and TfR, without compromising the barrier function. The effect of hypoxia was mediated by cellular signaling processes initiated by HIF-1α translocation from the cytosol to the nucleus. Our findings agreed with the observation that a tight brain endothelium with functional BBB properties is present early during the development of the brain vasculature when the microenvironment within the developing brain is hypoxic. Moreover, we observed that exposing confluent and functional cell monolayers to hypoxia led to enhancement of the barrier tightness.

In conclusion, the study reveals that the hypoxia may be beneficial for the generation of a mature brain endothelial phenotype, which could be a part of the explanation of why functional capillaries are formed in the environment in the early neuroectoderm during embryogenesis.

## Supplementary Information

**Additional file 1: Table S1**. Overview of the primer pairs used in this study. Table S2. Primary and secondary antibodies used in immunocytochemistry and Western blot analysis. **Figure S1**. Activities of GLUT-1 and LAT-1 in bovine brain capillary endothelial cells (BCEC) differentiated under normoxic and hypoxic conditions. A) The luminal uptake of [3H]-glucose in the presence and absence of 2 mM cold glucose and sodium in cells cultured under hypoxic conditions. The uptake data are shown as mean ± SEM of three individual cell passages of duplicates (n=3, total N=9). B) The luminal uptake of L-[4,5-3H]-leucine into endothelial cells was examined in the presence and absence of 100 μM BCH. The uptake data are shown as mean ± SEM of three individual cell passages of duplicates (n=3, total N=6). The donor concentrations of [3H]-glucose and L-[4,5-3H]-leucine were 20 and 9.5 nM, respectively. **Figure S2**. Expression of active β-catenin in BCECs cultured under normoxic and hypoxic conditions. A) Immunoblots of β-catenin in normoxic (N) and hypoxic (H) cells on the experimental day (n=3, total N=9). B) Immunocytochemical characterization of β-catenin in normoxic and hypoxic cells. Cells were stained after 10 min to 72 h upon seeding on permeable filter supports. All samples were counterstained with propidium iodide (red) to visualize cell nuclei. Scale bars = 50 μm. Images are representative of three individual experiments in triplicate (n=3, total N=9).

## Data Availability

The datasets used and/or analysed during the current study are available from the corresponding author on reasonable request.

## Abbreviations

BBB: Blood–brain barrier
BCEC: Brain capillary endothelial cells
Cl-5: Claudin-5
GLUT-1: Glucose transporter 1
HIF-1: Hypoxia-inducible factor-1
LAT-1: Large neutral amino acid transporter 1
P-gp: P-glycoprotein
TEER: Transendothelial electrical resistance
TfR: Transferrin receptor
TJ: Tight junction
