# Supplementary material for: Hypoxia increases expression of selected blood–brain barrier transporters GLUT-1, P-gp, SLC7A5 and TFRC, while maintaining barrier integrity, in brain capillary endothelial monolayers
Source: Fluids Barriers CNS. 2022 Jan 4;19:1. doi: 10.1186/s12987-021-00297-6 (PMC8725498; doi:10.1186/s12987-021-00297-6)
Supplement: Supplementary file 1 — Additional file 1: Table S1. Overview of the primer pairs used in this study. Table S2. Primary and secondary antibodies used in immunocytochemistry and Western blot analysis. Figure S1. Activities of GLUT-1 and LAT-1 in bovine brain capillary endothelial cells (BCEC) differentiated under normoxic and hypoxic conditions. A) The luminal uptake of [3H]-glucose in the presence and absence of 2 mM cold glucose and sodium in cells cultured under hypoxic conditions. The uptake data are shown as mean ± SEM of three individual cell passages of duplicates (n=3, total N=9). B) The luminal uptake of L-[4,5-3H]-leucine into endothelial cells was examined in the presence and absence of 100 μM BCH. The uptake data are shown as mean ± SEM of three individual cell passages of duplicates (n=3, total N=6). The donor concentrations of [3H]-glucose and L-[4,5-3H]-leucine were 20 and 9.5 nM, respectively. Figure S2. Expression of active β-catenin in BCECs cultured under normoxic and hypoxic conditions. A) Immunoblots of β-catenin in normoxic (N) and hypoxic (H) cells on the experimental day (n=3, total N=9). B) Immunocytochemical characterization of β-catenin in normoxic and hypoxic cells. Cells were stained after 10 min to 72 h upon seeding on permeable filter supports. All samples were counterstained with propidium iodide (red) to visualize cell nuclei. Scale bars = 50 μm. Images are representative of three individual experiments in triplicate (n=3, total N=9). [file 12987_2021_297_MOESM1_ESM.pdf]

Supplementary materials for:

Hypoxia increases expression of blood-brain barrier transporters SLC2A1, SLC7A5 and TFRC, while maintaining barrier integrity, in brain capillary endothelial monolayers.

Authors;

Burak Ozgür[1], Hans Christian Cederberg Helms[1], Erica Tornabene[1] & Birger Brodin[1]

1. Department of Pharmacy, University of Copenhagen, Universitetsparken 2, DK-2100, Copenhagen, Denmark

   *Corresponding author:
   Birger Brodin, Section of Pharmaceutical Design and Drug Delivery, Department of Pharmacy, University of Copenhagen, Universitetsparken 2, DK-2100, Copenhagen, Denmark Email;
   birger.brodin@sund.ku.dk
   Voice: +453533616

Keywords: Angiogenesis, hypoxia, low oxygen tension, endothelial cells, blood-brain barrier, tight junctions, brain

Table S1. Overview of the primer pairs used in this study.

Gene symbol	Gene name	PCR efficiency	Product Size (bp)	Primer Sequence (5' to 3')
HPRT1	Hypoxanthine guanine phosphoribosyl transferase 1	2.03	144	F: CGTGGTGATTAGCGATGATG<br>R: TTCATCACATCTCGAGCCAG
YWHAZ	Tyrosine 3-monooxygenase/tryptophan 5-monooxygenase activation protein zeta	1.90	100	F: AGCTGGTACAGAAGGCCAAA<br>R: TTGGATAATTCAGCTCCTTGC
SDHA	Succinate dehydrogenase complex	1.92	105	F: CTTCAAGGAGAGGGTTGACG<br>R: TCAACGTAGGAGAGCGTGTG
CLDN5	Claudin-5	2.06	122	F: CAGAAGTACGAGCTGGGAGC<br>R: TACTTCACCGGGAAGCTGAA
ABCB1	P-glycoprotein (Pgp)	1.99	127	F: CGGGACAGAAAGCTCAGTTC<br>R: TAATGGCGCAAAATACACCA
SLC2A1	Glucose transporter 1 (GLUT-1)	1.92	139	F: TACCCCAAGAGGTGGCTATG<br>R: CTGGTCTCAGGCAAGGAAAG
SLC7A5	L-type amino acid transporter 1 (LAT-1)	1.91	111	F: TAGCCAATCTGGATCCCAAG<br>R: TCAAGTAATTCCATCCCCCA
FLT1	Vascular endothelial growth factor receptor 1 (VEGFR1)	1.91	292	F: CCTGCTTCCAAGAACAAAGC<br>R: CTGTTGTCTCGCAGGTCAAA

Table S2. Primary and secondary antibodies used in immunocytochemistry and Western blot analysis.

Target protein	Clonality	Host	Dilution factor	Supplier and catalogue ID
Primary antibodies				
HIF-1α	Monoclonal	Rabbit	1:1000	Cell Signaling (36169T)
von Willebrand factor	Polyclonal	Rabbit	1:250	Abcam (ab6994)
Claudin-5	Polyclonal	Rabbit	1:200	Abcam (ab15106)
Zonula occludens-1	Polyclonal	Rabbit	1:100	Abcam (ab59720)
Glut-1	Polyclonal	Rabbit	1:100	Thermo Scientific (pa1-1063)
LAT-1	Polyclonal	Rabbit	1:100	Bioss Antibodies (bs-10125R)
P-glycoprotein	Polyclonal	Rabbit	1:100	Abcam (ab129450)
For WB: P-glycoprotein	Polyclonal	Rabbit	1:500	Kindly provided by Lundbeck
Transferrin receptor	Monoclonal	Mouse	1:100	Thermo Scientific 13-6800
β-catenin (active)	Monoclonal	Rabbit	1:100	Cell Signaling (19807T)
β-actin	Monoclonal	Mouse	1:2500	Sigma-Aldrich (A5441)
Secondary antibodies conjugated with Alexa-488 or HRP				
Anti-rabbit	Polyclonal	Goat	1:7000	Molecular Probes (G-21234)
Anti-mouse	Polyclonal	Goat	1:4500	Invitrogen (62-6520)
Anti-rabbit	Polyclonal	Goat	1:200	Life Technologies (AV1008)

Anti-mouse	Polyclonal	Goat	1:200	Invitrogen (AH017)

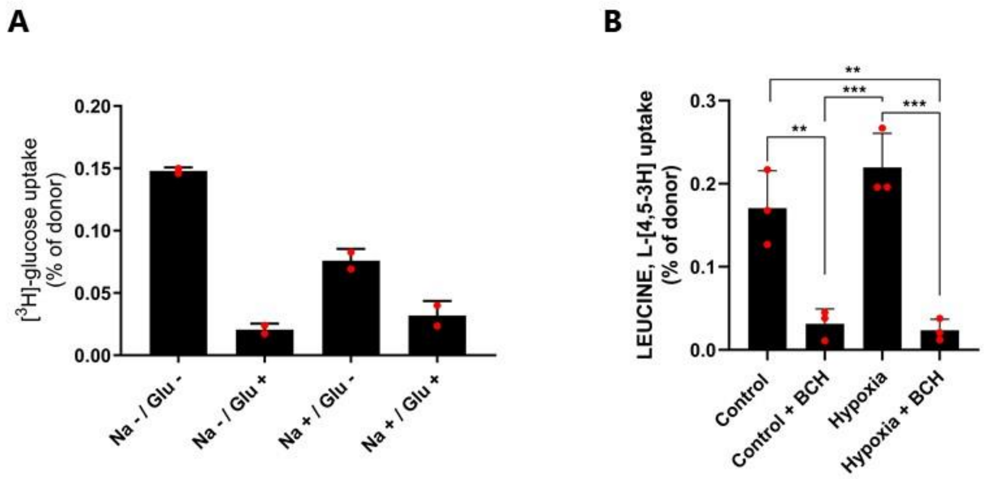

Figure S1. Activities of GLUT-1 and LAT-1 in bovine brain capillary endothelial cells (BCEC) differentiated under normoxic and hypoxic conditions. A) The luminal uptake of [3H]-glucose in the presence and absence of 2 mM cold glucose and sodium in cells cultured under hypoxic conditions. The uptake data are shown as mean ± SEM of three individual cell passages of duplicates (n=3, total N=9). B) The luminal uptake of L-[4,5-3H]-leucine into endothelial cells was examined in the presence and absence of 100 μM BCH. The uptake data are

shown as mean ± SEM of three individual cell passages of duplicates (n=3, total N=6). The donor concentrations of [3H]-glucose and L-[4,5-3H]-leucine were 20 and 9.5 nM, respectively.

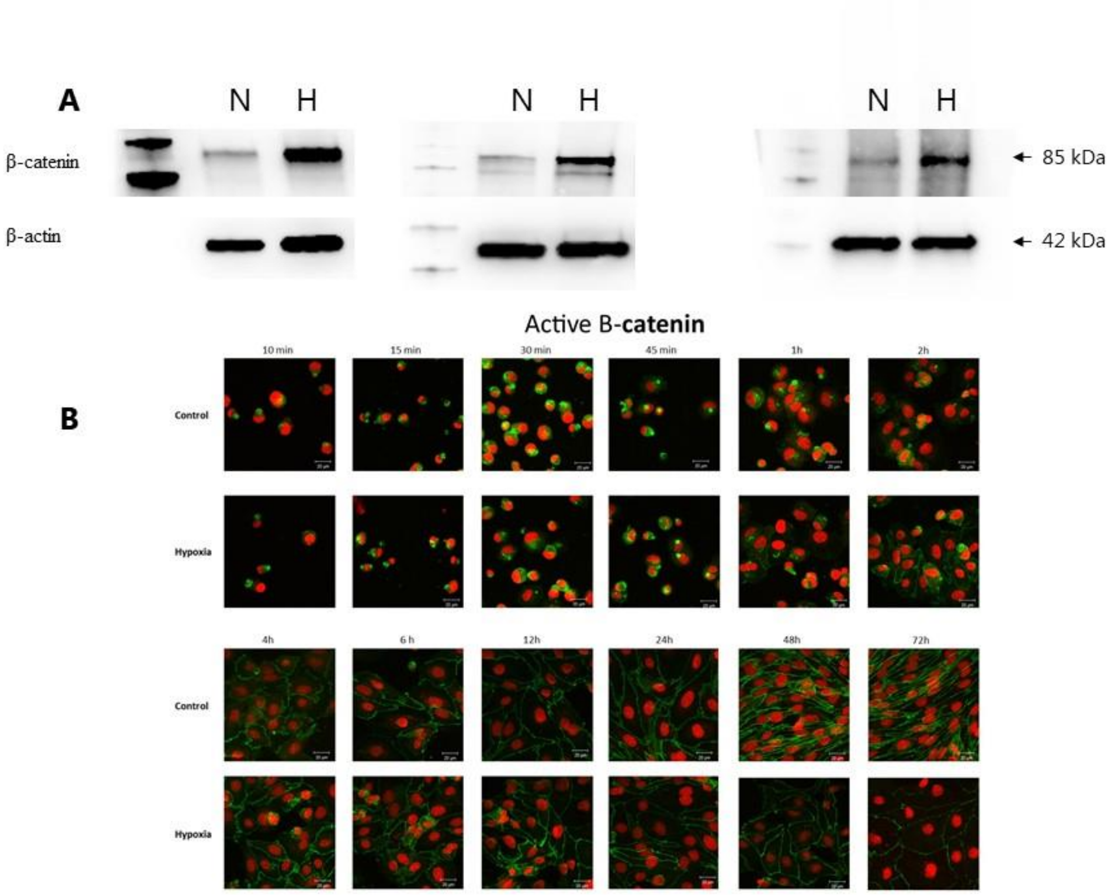

Figure S2. Expression of active β-catenin in BCECs cultured under normoxic and hypoxic conditions. A) Immunoblots of β-catenin in normoxic (N) and hypoxic (H) cells on the experimental day (n=3, total N=9). B) Immunocytochemical characterization of β-catenin in normoxic and hypoxic cells. Cells were stained after 10 min to 72 h upon seeding on permeable filter supports. All samples were counterstained with propidium iodide (red) to visualize cell

nuclei. Scale bars = 50 μm. Images are representative of three individual experiments in triplicate (n=3, total N=9).
